# Supplementary figures and images for: AXR1 affects DNA methylation independently of its role in regulating meiotic crossover localization
Source: PLoS Genet. 2020 Jun 29;16(6):e1008894. doi: 10.1371/journal.pgen.1008894 (PMC7351236; doi:10.1371/journal.pgen.1008894)

# Christophorou\_S1 Fig

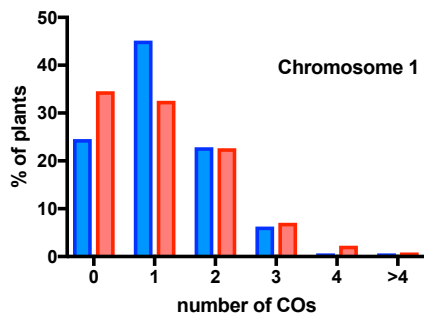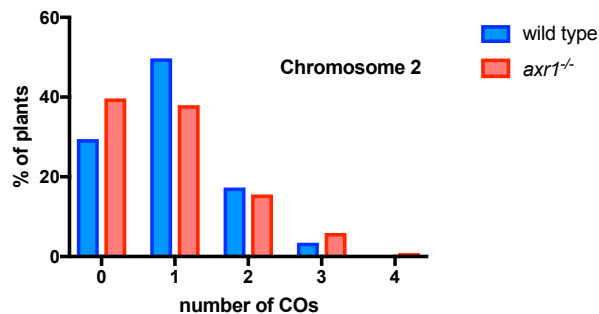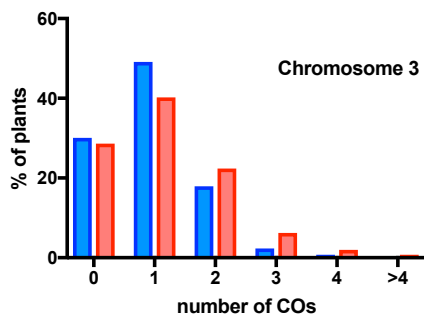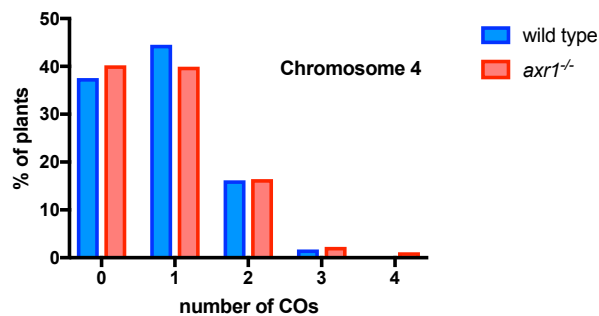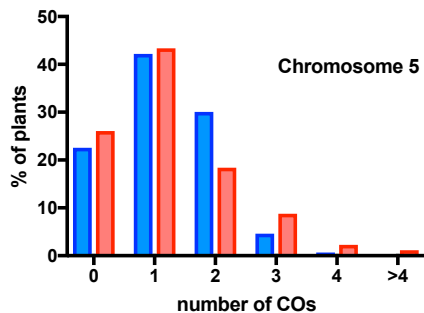

Supplement: S1 Fig — (PDF) [file pgen.1008894.s001.pdf]

## subtelomeric regions

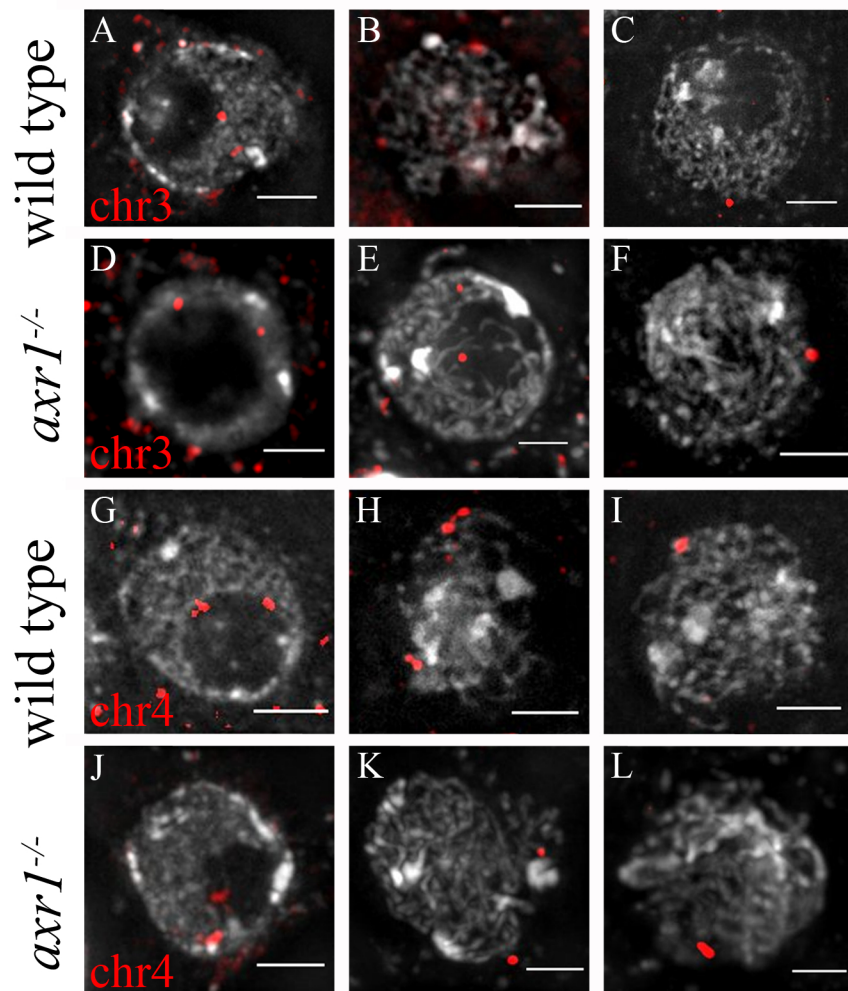

## proximal regions

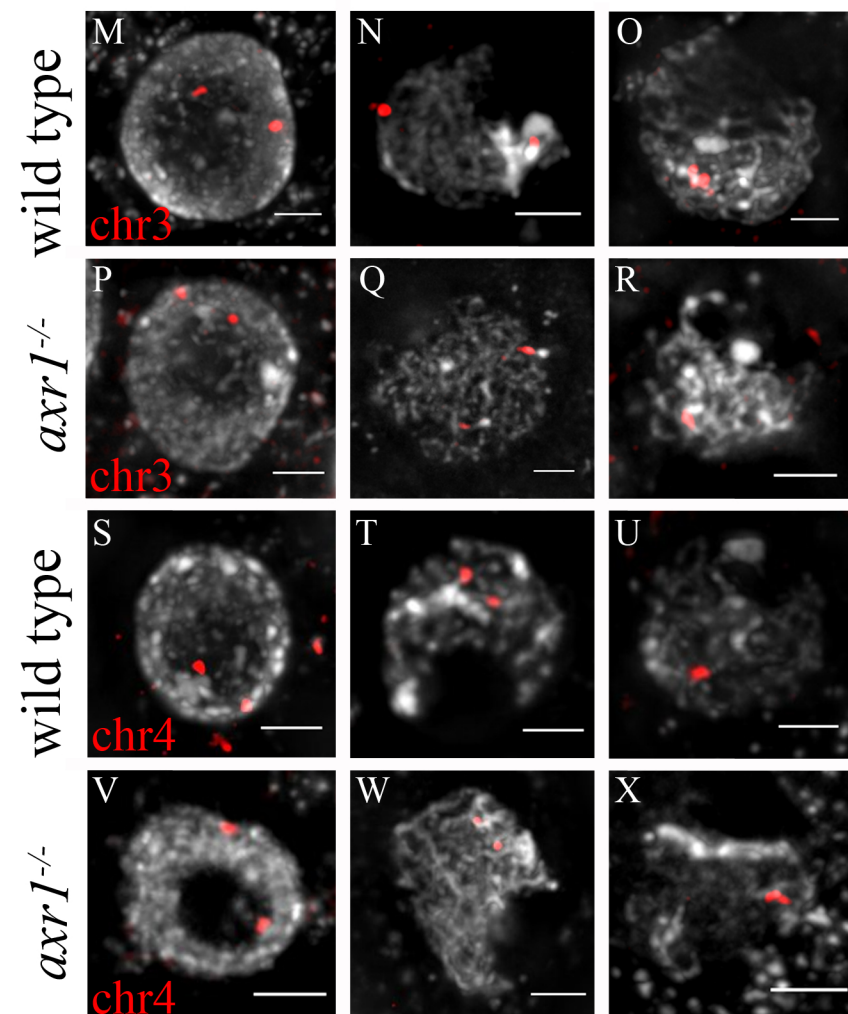

Supplement: S2 Fig — (PDF) [file pgen.1008894.s002.pdf]

## Slide 1
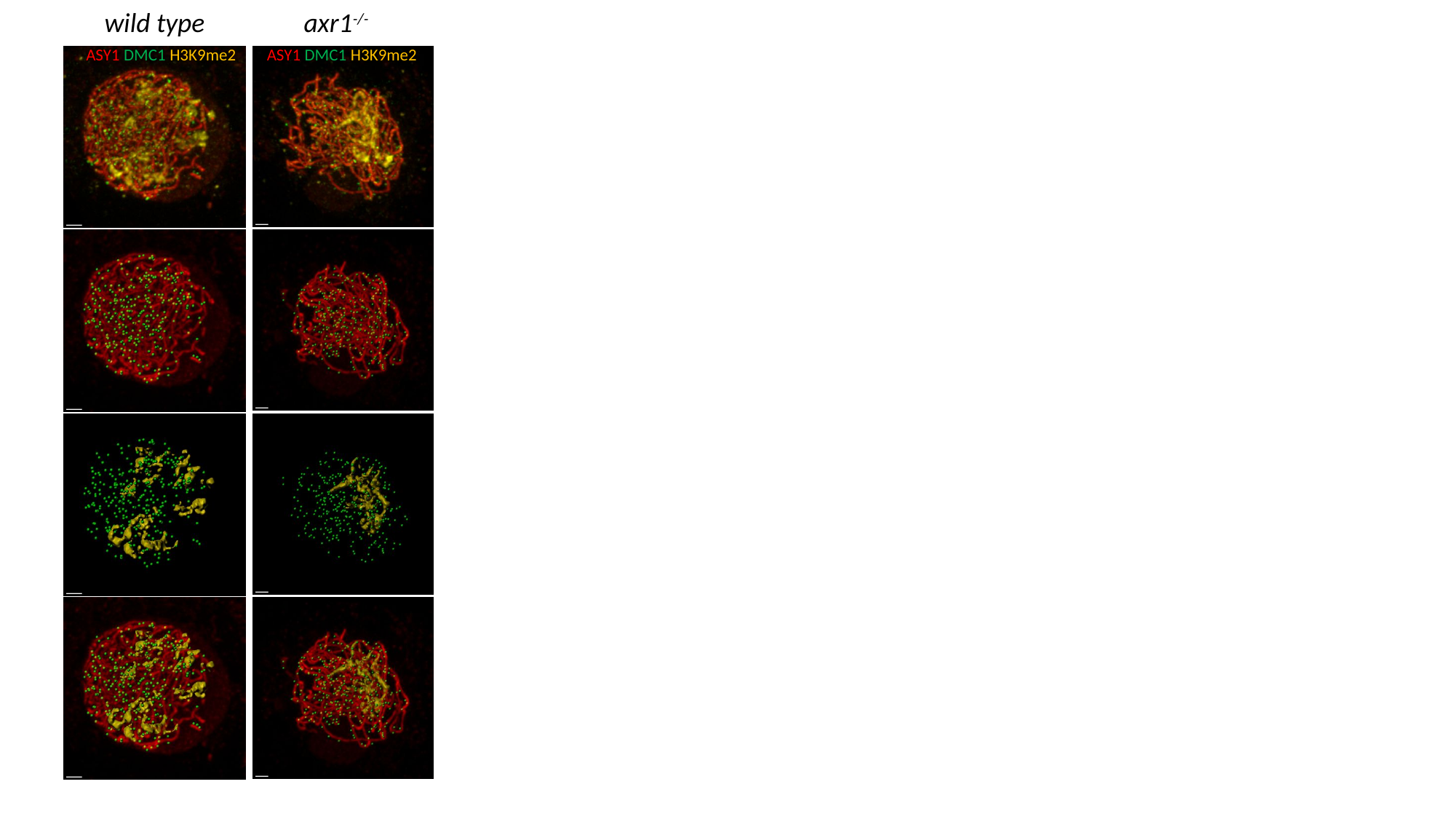

wild type
axr1-/-
ASY1 DMC1 H3K9me2
ASY1 DMC1 H3K9me2

Supplement: S3 Fig — (PPTX) [file pgen.1008894.s003.pptx]

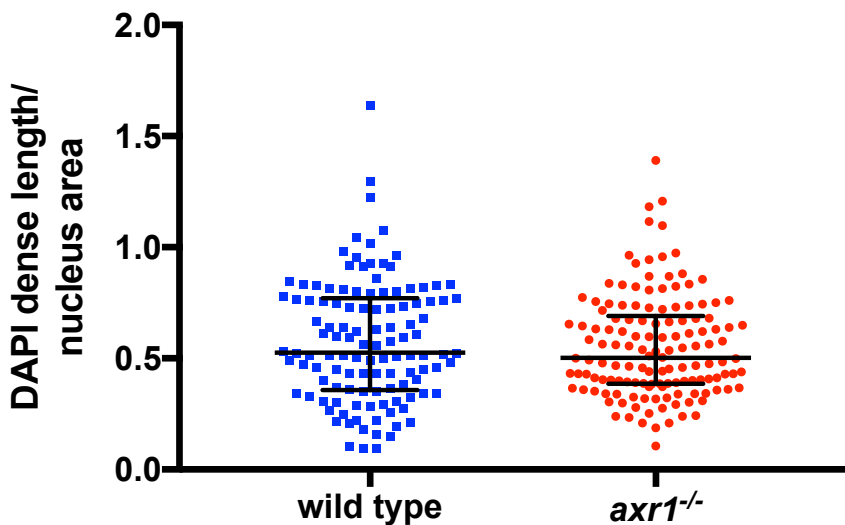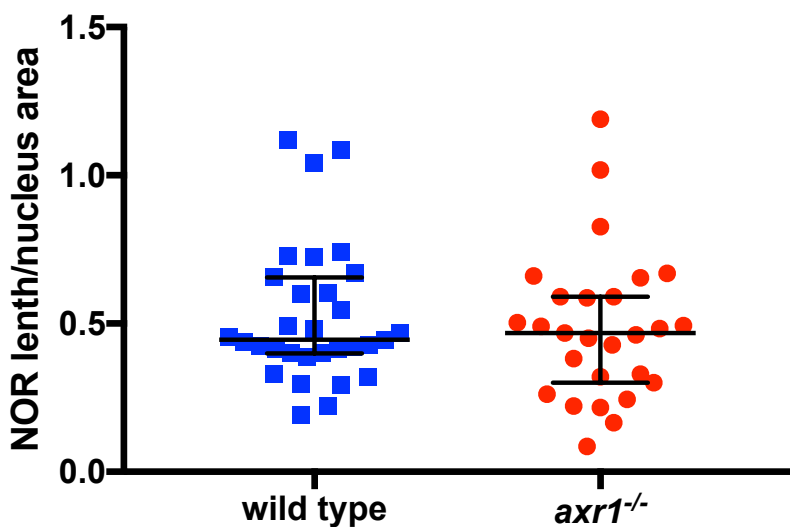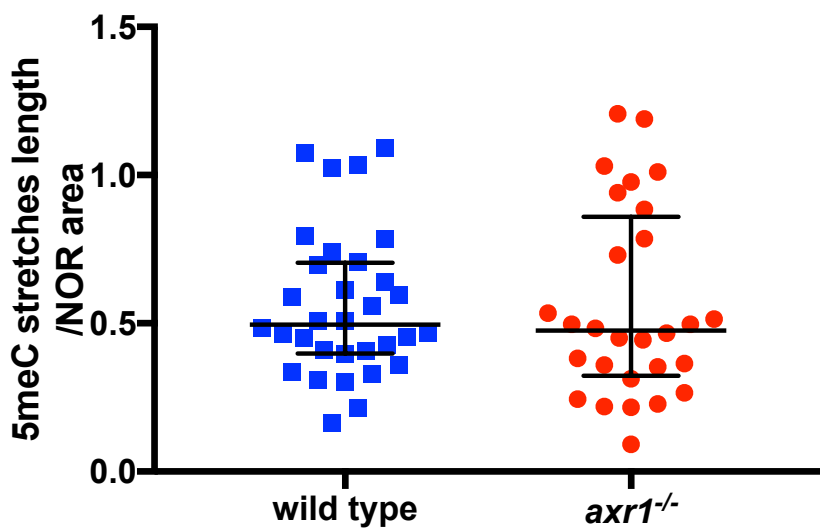

Supplement: S4 Fig — (PDF) [file pgen.1008894.s004.pdf]

Sup Figure S5

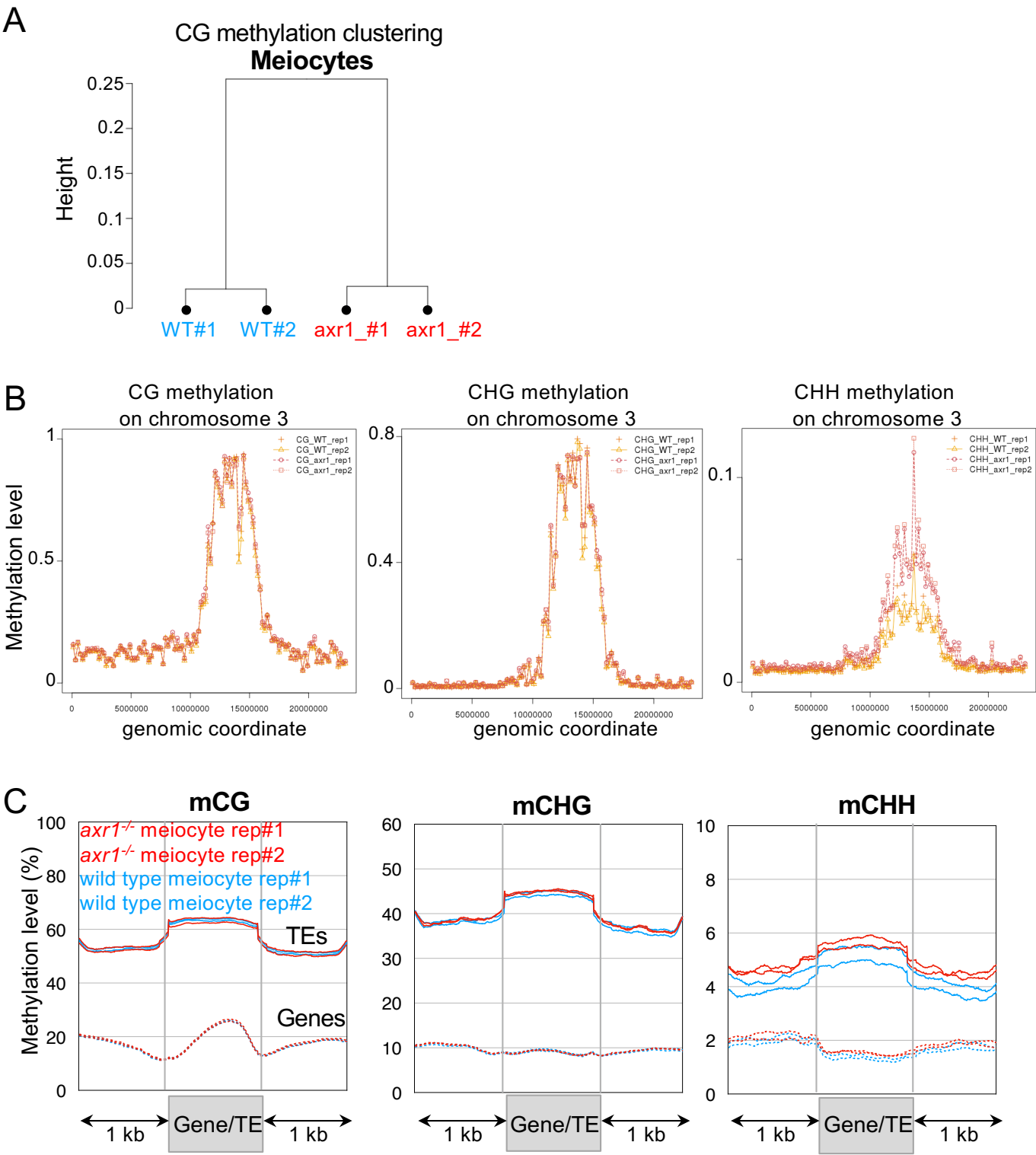

Supplement: S5 Fig — (PDF) [file pgen.1008894.s005.pdf]

Sup Figure S6

**A** CG methylation clustering  
**Somatic cells**

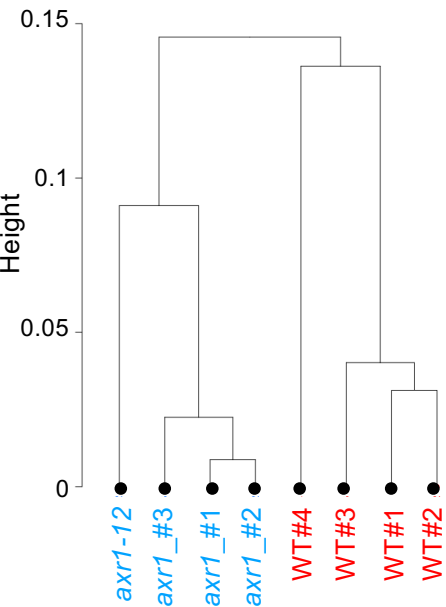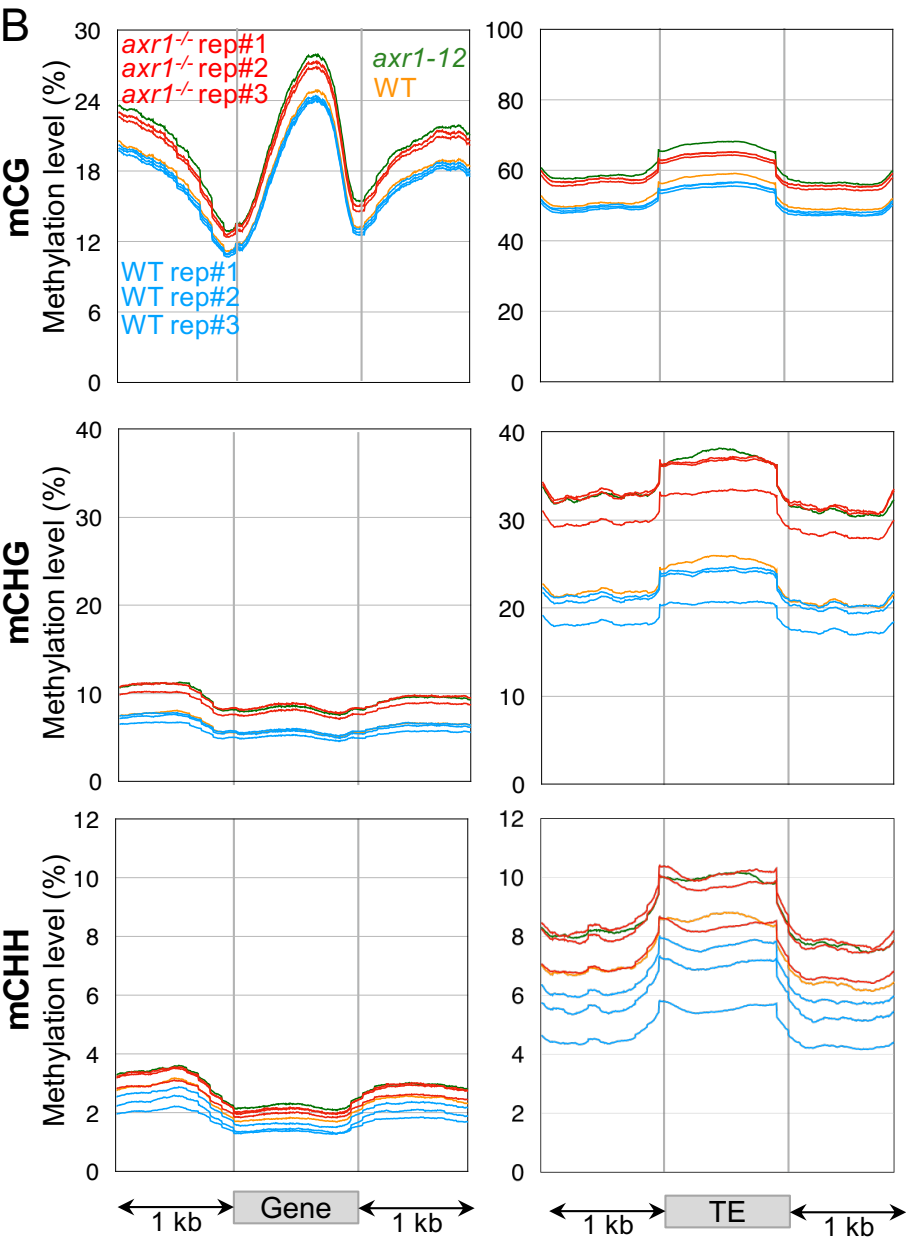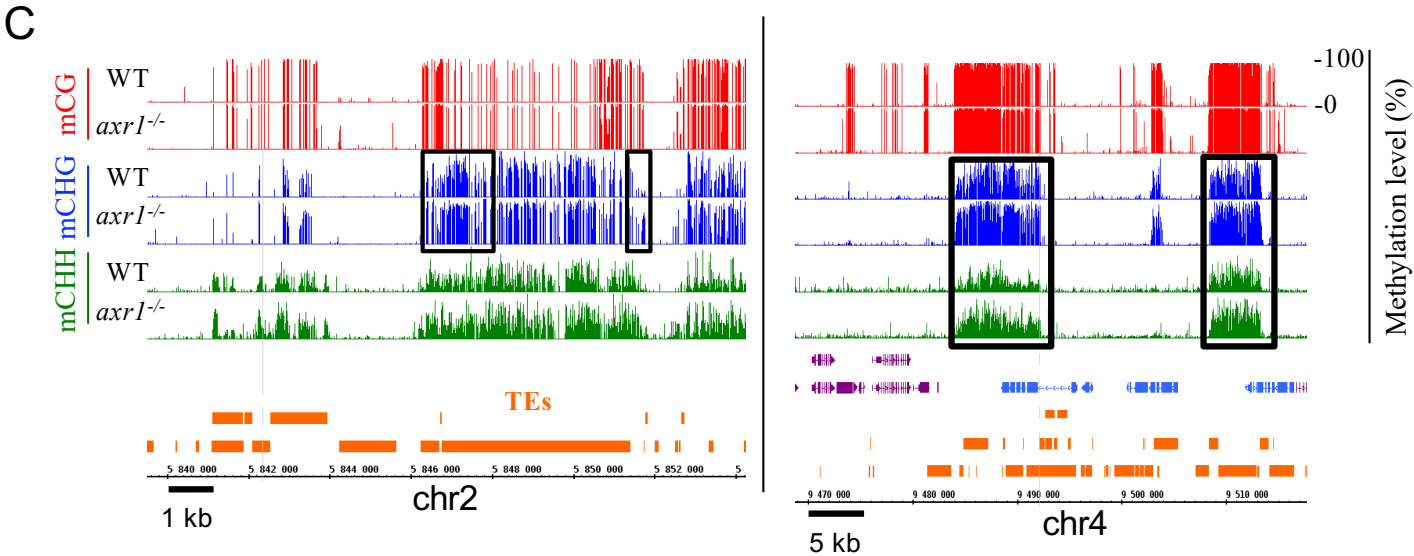

Supplement: S6 Fig — (PDF) [file pgen.1008894.s006.pdf]

Sup Figure S7

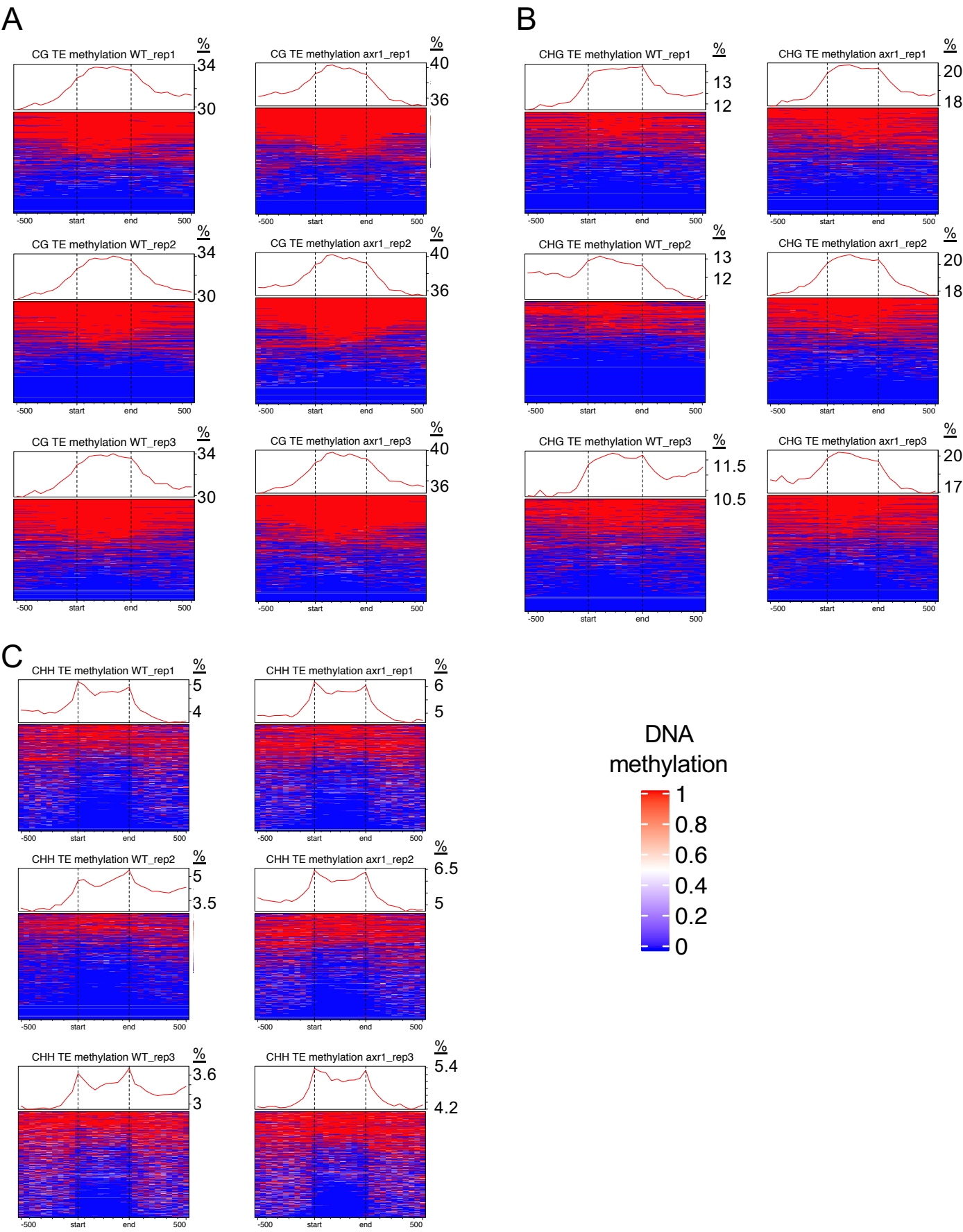

Supplement: S7 Fig — (PDF) [file pgen.1008894.s007.pdf]

Sup Figure S8

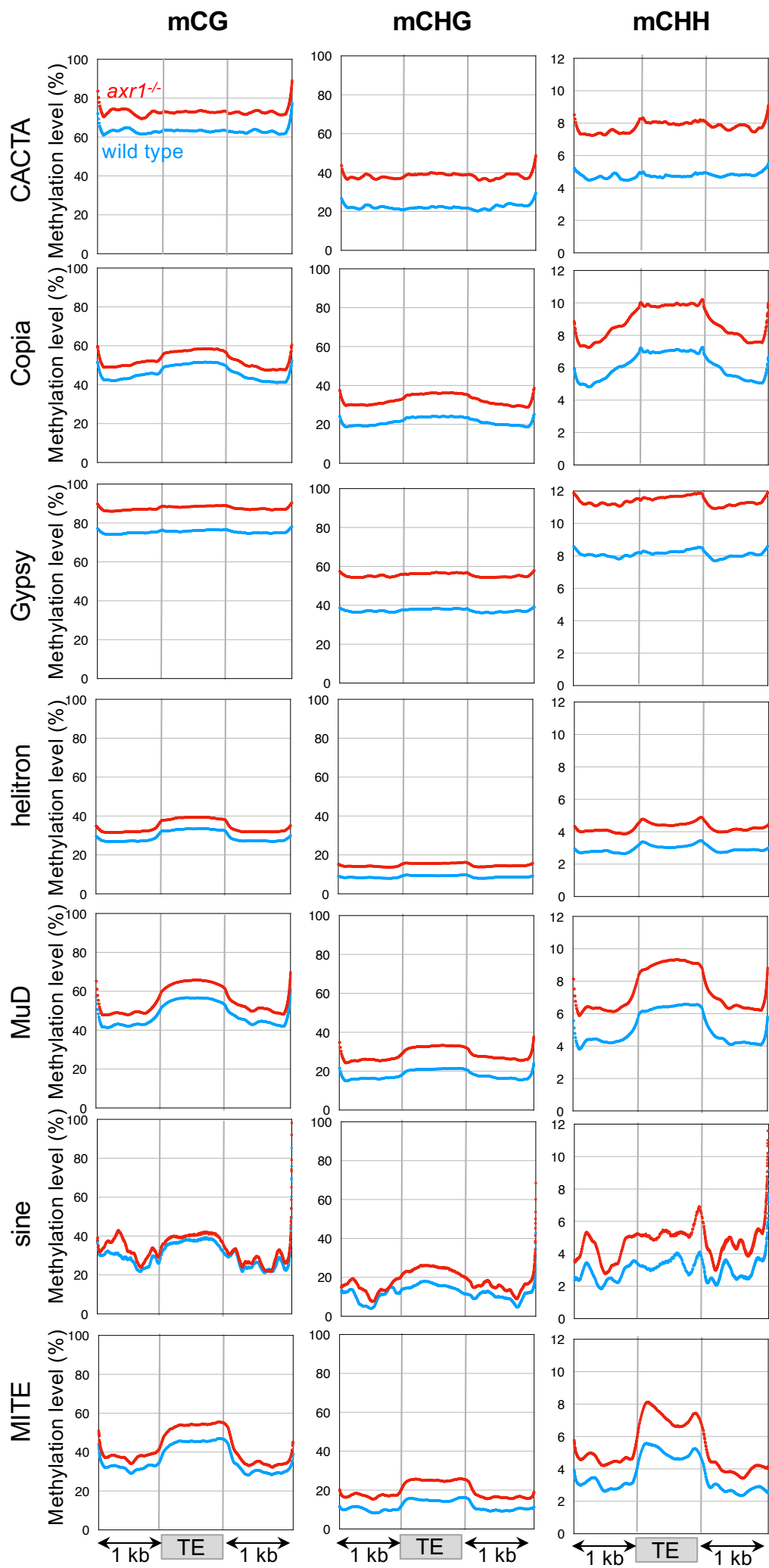

Supplement: S8 Fig — (PDF) [file pgen.1008894.s008.pdf]

Sup Figure S9

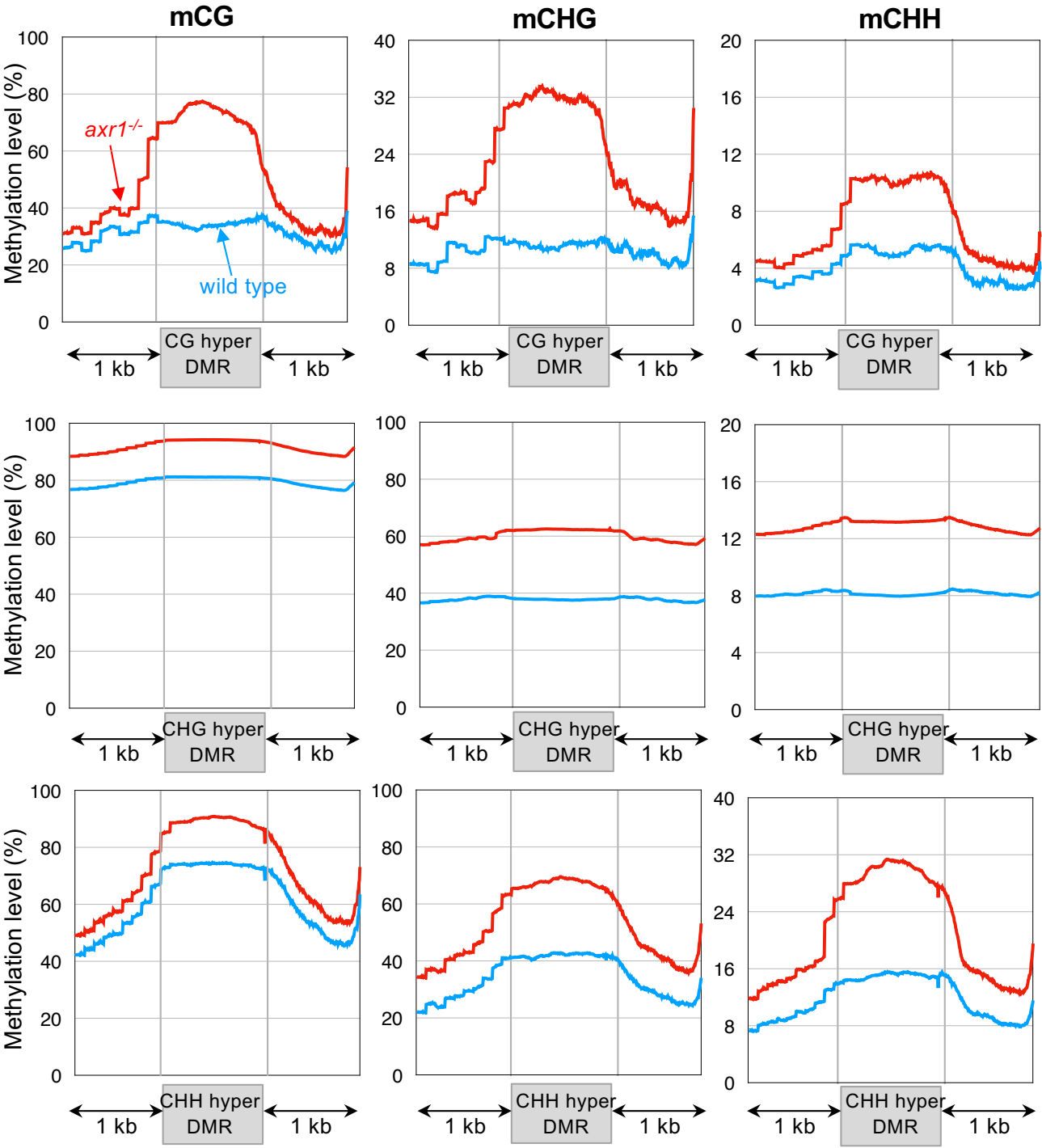

Supplement: S9 Fig — (PDF) [file pgen.1008894.s009.pdf]

Sup Figure S10

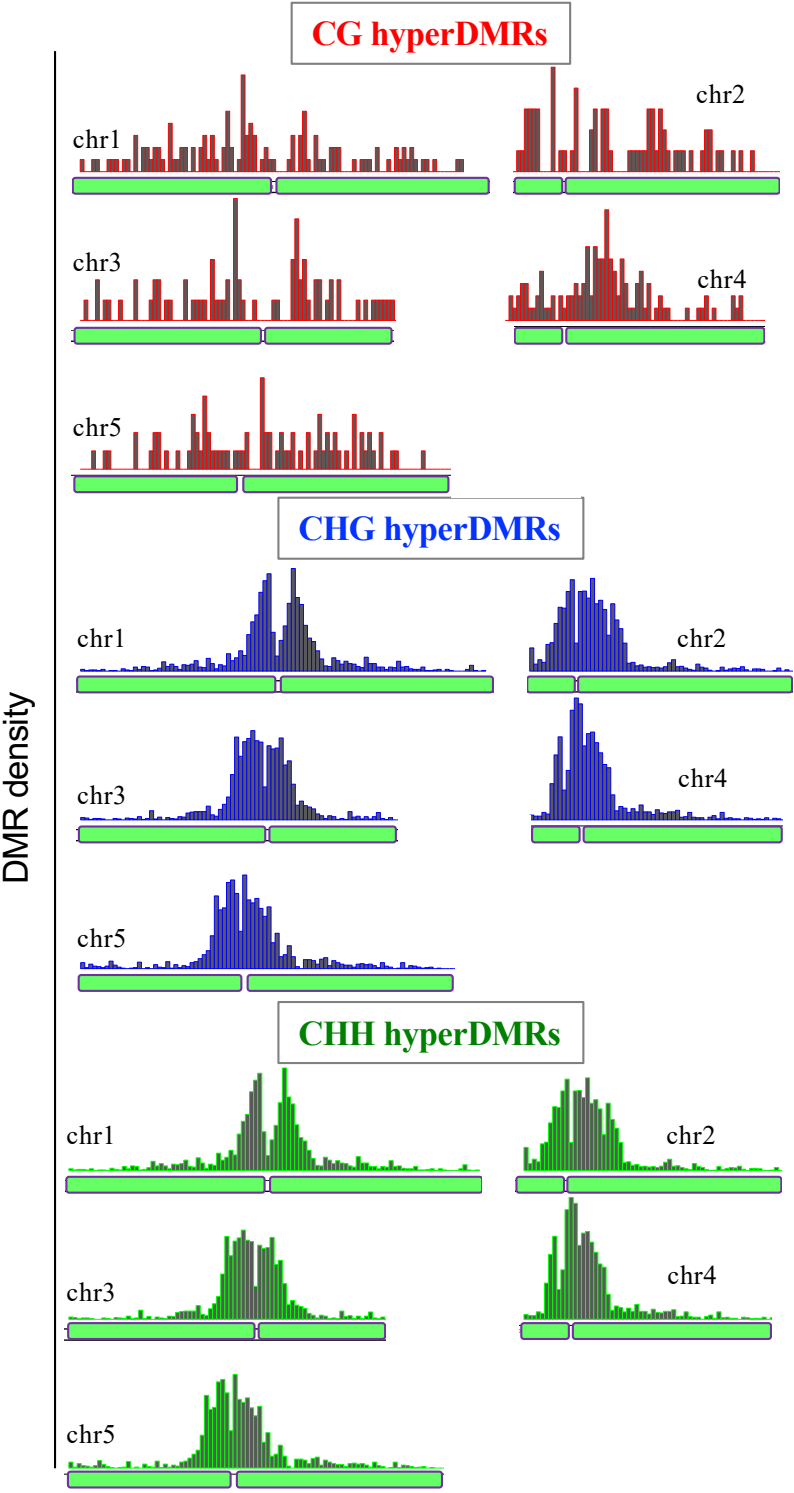

Supplement: S10 Fig — (PDF) [file pgen.1008894.s010.pdf]

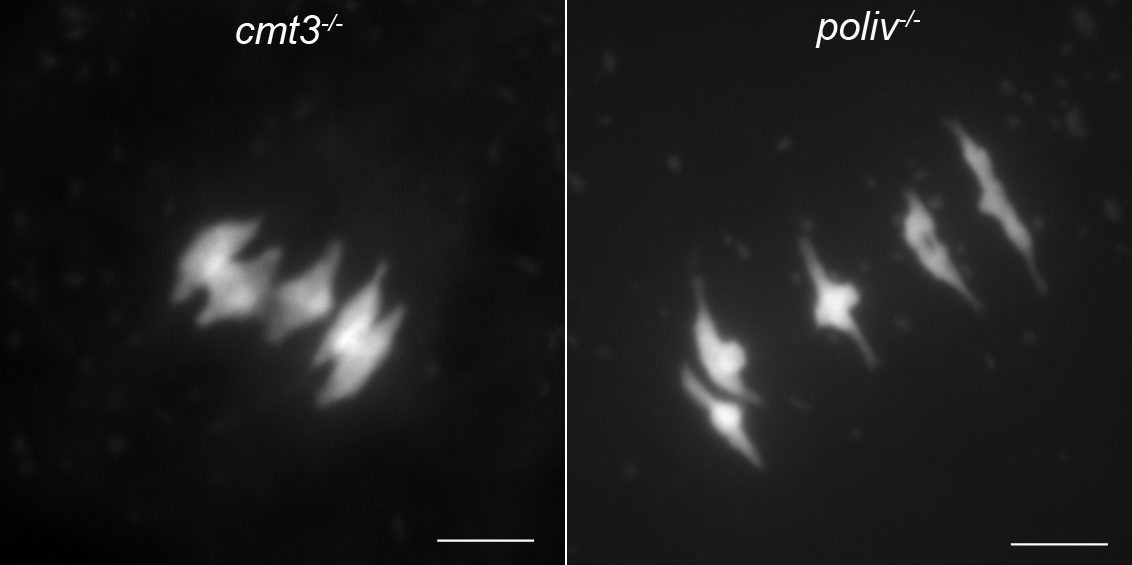

Supplement: S11 Fig — (TIF) [file pgen.1008894.s011.tif]
