# Supplementary material for: AXR1 affects DNA methylation independently of its role in regulating meiotic crossover localization
Source: PLoS Genet. 2020 Jun 29;16(6):e1008894. doi: 10.1371/journal.pgen.1008894 (PMC7351236; doi:10.1371/journal.pgen.1008894)
Supplement: Attachment — Submitted filename: Christophorou _responsetoreviewers_26052020.pdf [file pgen.1008894.s019.pdf]

Reviewer #2

This revision provided additional data, in the form of key controls, that strengthen the authors claims regarding the effects of axr1 mutants on DNA methylation patterns. Regarding this new data I have several additional comments as detailed below. Overall, as was mentioned by another reviewer, the data are now more clear, but the messaging at times could be more transparent about the connections (or lack thereof) between the different phenotypes observed in the *axr1* mutant. I have provided feedback related to this point as well.

DNA methylation analysis:

1. In Table S2, please make it clear which samples and controls were grown and prepped together. In the response to reviewers, it is stated that axr1-12 and presumably WT rep#4 were done in parallel, but this is not clear in the table.
   Table S2 has been changed to indicate which samples were sequenced with their respective controls. Indeed, *axr1-12* and WT rep#4 were grown at the same time and sequenced together.
2. In Figure 6A, please add the WT control for axr1-12. Also, please clarify why this data is shown as a boxplot rather than a metaplot to match the other panels in the figure.
   The WT control for *axr1-12* is now included in Fig 6A. Metaplots give information regarding a specific region and since MET1 controls CG methylation in both genes and TEs, boxplots showing the CG methylation levels genome-wide seem to be more appropriate for CGs.
   CHG and CHH methylation differences are more adequately represented by showing regions specifically targeted by these methylation types, namely TEs/RdDM targets.
3. In Figure S5A please add the WT control and axr1-12 to the clustering analysis.
   Both WT control and *axr1-12* were added to the clustering analysis. Consistently, all WT samples and all *axr1* samples respectively cluster together. Fig S5 and S6 were reorganized to have one figure dedicated to meiocytes and the other one to plants.
4. In Figure 4A, metaplots are used to show the differences between the mutant and WT methylation levels across the chromosomes, where it is clear the hyper CHH is in the centromeric regions, but in Fig 5a differences are shown with scatter plots. For consistency it might be useful to represent this data from two different tissues in the same manner, or show both types of plots even if some of the data is in the supplement.
   We agree and we have homogenized both Fig 4 and 5 to represent DNA methylation in the same way. Metaplots are now used for meiocytes (Fig 4A) and somatic cells (Fig 5A).
5. It is standard practice to compare the methylation patterns of two alleles at a finer scale than presented here. Based on the metaplot analyses, both axr1 and axr1-12 show hypermethylation around the centromeres, but do they show hypermethylation at a similar set of genomic loci? Please identify DMRs in the axr1-12 mutant and provide a comparison with those identified for axr1. Assuming there is a high degree of overlap the methylation patterns, the WT and axr1-12 tracks could be added to the screen shots shown in Fig S5C.
   DMRs obtained between *axr1*$^{-/-}$ and the WT rely on three biological replicates for both genotypes, while *axr1-12* and the corresponding WT control were sequenced only once. Doing a proper DMR analyses of *axr1-12* would require making new sequencing libraries and another round of sequencing. Since we show that the *axr1-12* patterns of methylation are very similar to those of *axr1*$^{-/-}$ genome-wide (Fig 6A) and for TEs (Fig S6), we believe that this is unnecessary.
6. Please reference and indicate in the methods what data sets were used to compare the axr1 DMRs to those in met1, cmt3, kyp, and poliv mutants.

The data sets were published by Stroud *et al*, Cell, 2013. This is now indicated in the methods.

Text comments:

1. Perhaps a title that more clearly conveys the conclusions of the work would be “AXR1 affects DNA methylation independent of its role in regulating meiotic crossover localization”?
   We agree and thank the reviewer for being so positive about our conclusion that the effect of *axr1* on DNA methylation is independent of that over the crossovers. The title has been changed accordingly.
2. The running title could also be modified to better reflect the data
   The running title has been modified to “AXR1, DNA methylation and CO control”
3. Page 2. Its not clear what by the statement that axr1 affects “general methylation” in a plant? What is general methylation? Please be more specific.
   We have changed “general methylation” to “DNA methylation”.
4. Several times the authors state the methylation changes as “massive” or “drastic”, but no value is provided. Such a statement should either be pared with an empirical value (i.e the average % increase in methylation over the WT) and a reference to other work showing this change is very large compared to other mutants, or such adjectives should be removed.
   We have removed the words “massive” or “drastic” wherever needed in the manuscript.
5. In the intro the authors conclude that AXR1 acts to limit DNA methylation pathways. The data do support this conclusion, but do not provide information on whether this is a direct or indirect effect of AXR1 function. The text should be modified to make this clear.
   Done
6. In the author summary, the last sentence mentions “methylation of nucleic acids”. Since other bases can be modified, but this work is specific to cytosine methylation, the text should be modified to make this clear.
   Done

Minor comments:

- Figure 1B is referenced out of order, please reorder the text or figure.
  Done
- In the main text, page 8 line 25, the authors refer to 15 intervals with different CO rates. On Fig 1C, I count 18 asterisks (*) and there are also appear to be 18 regions in table S1. Please clarify.
  Done
- As the text referring to Fig 1B uses Mb units rather that % of chromosome length, perhaps the graph could be replotted or the text edited to allow this figure to be more easily interpreted.
  % of chromosomes have been added
- The Y axis label for Fig 2B is cut-off.
  Changed
- In Figure S6 I believe the data is from meiocytes based on the legend, but they are labeled as “sc” which I presume stands for somatic cells. Please clarify.
  “sc” stands for “single cell”. This has been changed to “meiocyte” in the figure.
- It is confusing that the supplemental data for the meiocyte and somatic tissues are mixed between Fig S5 and S6. I might be helpful to group the meiocyte and somatic data into separate

figures. Indeed, as there are just two replicates and the metaplots are not too complicated perhaps fig S6 could be removed and in Fig 4 both replicates cold be shown?
We agree. Now, Fig S5 is dedicated specifically to meiocyte data and Fig S6 is for plants. For consistency, Fig S5 and S6 were reordered and switched as meiocyte data appear first in the text.

- In Fig S7 several of the methylation scale bars for the heatmaps are overlapping the Y axis labels for the metaplots. Also, please indicate in the legend if the order of TEs is the same for the all the CG, CHG, and CHH heatmaps or whether they were ordered separately based on a specific parameter in each heatmap.
Fig S7 was improved and overlapping scale bars were removed. Those are enriched heatmaps showing the most methylated TEs first. This is now indicated in the legend.
- I could not find Fig 7 or Fig S1-3 in the revised version. I assume they are not changed from the first revision. If otherwise, please clarify.
They were in the PDF file built by the Plos Genetics system and indeed they were and they are unchanged from the previous version

Reviewer #3:

I'm really happy that the authors included DNA methylation analysis of axr1 -/- meiocytes in the revised manuscript. These data, however, introduce a major twist in how the link between DNA methylation and CO patterns in axr1 -/-, if any can be interpreted.
The authors now show that cytosine methylation in the CG and CHG contexts is not altered in axr mutant meiocytes. They found, however, a change in CHH methylation. Although the level of CHH methylation is relatively low, compared to CG and CHG methylation, the relationship between CHH methylation on its own and recombination has not been studied. Hence, it cannot be excluded that the altered CO distribution in axr1 -/- is actually caused by the CHH methylation level change.

Furthermore, since the new data indicate misalignment between meiocyte and leaf DNA methylation, the effect of double mutants between axr1 and met1, cmt3, as well as poliv on DNA methylation patterns in meiosis remains unclear.

Given all this, the conclusions on CO distribution being uncoupled from DNA methylation in axr1$^{-/-}$ (page 3, page 12, page 13, page 16) are not well supported. Perhaps it would be better just to focus the manuscript on DNA methylation?

We thank the reviewer for pointing out the "misalignment". We agree that we did not explain this well in the manuscript. Therefore, accordingly, we have added a paragraph on Page 10 in the results section.

"We have previously shown that CG and CHG methylation are strongly reinforced in the germ cells (including the meiocyte) in comparison to somatic tissues (31,48,49). Indeed, CG and CHG methylation in wild-type (and *axr1*$^{-/-}$) meiocytes mimic those in *axr1*$^{-/-}$ leaves (Fig 4B and 5B). In other words, although *axr1* mutation increases CG and CHG methylation in soma (Fig 5B), it

cannot further increase CG and CHG methylation in meiocyte, whose methylation levels are already as high as *axr1*$^{-/-}$ leaves (Fig 4B and 5B). This result suggests that *axr1* mutation affects general methylation in the plant, rather than specifically affecting the methylation in meiocytes. Consistent with this idea, in the CHH context, where meiocytes have lower CHH methylation compared to leaves (Fig 4B, 5B) (ref 31), *axr1* mutation induces CHH hypermethylation in meiocytes (Fig. 4B). Taken together, our results show that the effect of *axr1* mutation on meiocyte methylome occurs prior to reproductive development, by affecting general DNA methylation pathways."

Regarding whether the CHH hypermethylation observed in meiocytes is responsible for the altered CO distribution, we think this is very unlikely. This is because, firstly, we have shown via whole-genome methylation profiling that methylation pathway mutations (eg. *met1*, *cmt3*, *pol iv*) are epistatic over *axr1* over the methylation phenotype (result section 7). Our data also demonstrates that regarding the CO phenotype, *axr1* is epistatic over these methylation pathway mutants (*met1*, *cmt3*, *pol iv*). Therefore, genetically, the methylation and CO phenotypes are uncoupled, except of course, if the methylation pathway mutations (ie. *met1*, *cmt3*, *pol iv*) do not affect meiocyte methylation. MET1 and CMT3 are maintenance methyltransferases without which methylation will be lost through cell divisions, therefore *met1* and *cmt3* mutant meiocytes would undoubtedly lose, respectively, CG and CHG methylation. Consistently, *cmt3* sperm, the haploid gamete produced by meiocytes, loses CHG methylation almost entirely, just like *cmt3* leaf (ref 38). Pol IV works in the RdDM pathway, whose defect we have shown to reduce CHH methylation in meiocytes as in leaves (ref 31; also see graph below). Similarly, RdDM mutations only have weak effects on one Interval spanning the centromere of chromosome 3 (Underwood CJ, Choi K, Lambing C, Zhao X, Serra H, Borges F, et al. Epigenetic activation of meiotic recombination near Arabidopsis thaliana centromeres via loss of H3K9me2 and non-CG DNA methylation. Genome Res. 2018;28(4):519–31). Altogether, we agree that ultimately it would be the best to isolate meiocytes from *met1 axr1*, *cmt3 axr1* and *pol iv axr1* double mutants to show that the CHH methylation is reduced. However, isolating meiocytes is very time consuming, especially at the current time of the pandemic, not to mention that *met1* (and to an extent *axr1*) single mutant is already quite sick for meiocyte extraction. Also, given our knowledge about how these methylation pathways work and published single mutant data, it is very unlikely that the meiocyte methylation is not reduced in double mutants.

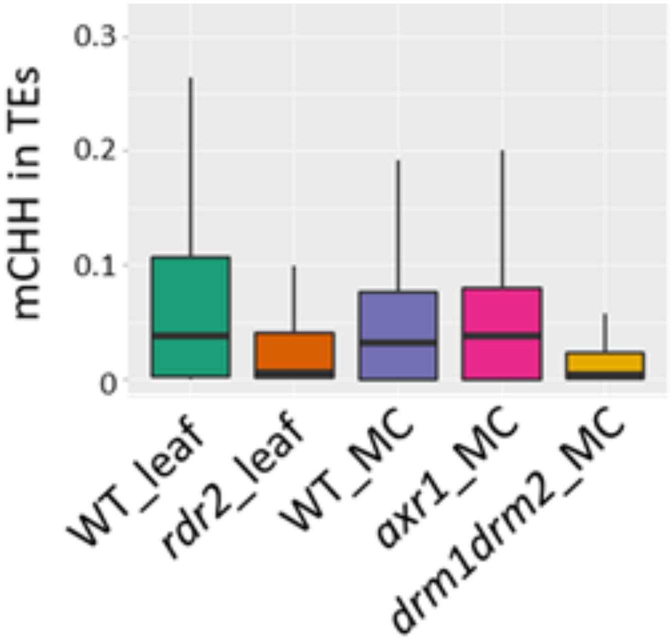

rdr2 and *drm1drm2*, RdDM mutants.

MC, meiocyte.

[A1]

Note: rdr2, and drm1/2 are both components of the RdDM pathway; MC, meiocyte.

Also, Figures S1, S2, and S3 are missing from the revised manuscript.

They were in the PDF file built by the Plos Genetics system
