# Supplementary material for: AXR1 affects DNA methylation independently of its role in regulating meiotic crossover localization
Source: PLoS Genet. 2020 Jun 29;16(6):e1008894. doi: 10.1371/journal.pgen.1008894 (PMC7351236; doi:10.1371/journal.pgen.1008894)
Supplement: Attachment — Submitted filename: Christophorou_ResponseToReviewers.docx [file pgen.1008894.s016.docx]

Dear Dr Mezard,

Thank you very much for submitting your Research Article entitled 'Meiotic Crossover localisation and DNA methylation are uncoupled In Arabidopsis thaliana when AXR1 is inactivated' to PLOS Genetics. Your manuscript was fully evaluated at the editorial level and by three independent peer reviewers. The reviewers appreciated the attention to an important problem but raised some substantial concerns about the current manuscript. Based on the reviews, we will not be able to accept this version of the manuscript, but we would be willing to review again a much-revised version. We cannot, of course, promise publication at that time.

Should you decide to revise the manuscript for further consideration here, your revisions should address the specific points made by each reviewer. We will also require a detailed list of your responses to the review comments and a description of the changes you have made in the manuscript.

If you decide to revise the manuscript for further consideration at PLOS Genetics, please aim to resubmit within the next 60 days, unless it will take extra time to address the concerns of the reviewers, in which case we would appreciate an expected resubmission date by email to [plosgenetics@plos.org](mailto:plosgenetics@plos.org).

If present, accompanying reviewer attachments are included with this email; please notify the journal office if any appear to be missing. They will also be available for download from the link below. You can use this link to log into the system when you are ready to submit a revised version, having first consulted our [Submission Checklist](http://journals.plos.org/plosgenetics/s/submit-now#loc-submission-checklist).

To enhance the reproducibility of your results, we recommend that you deposit your laboratory protocols in [protocols.io](http://protocols.io), where a protocol can be assigned its own identifier (DOI) such that it can be cited independently in the future. For instructions see our [guidelines](http://journals.plos.org/plosgenetics/s/submission-guidelines#loc-materials-and-methods).

Please be aware that our [data availability policy](http://journals.plos.org/plosgenetics/s/data-availability) requires that all numerical data underlying graphs or summary statistics are included with the submission, and you will need to provide this upon resubmission if not already present. In addition, we do not permit the inclusion of phrases such as "data not shown" or "unpublished results" in manuscripts. All points should be backed up by data provided with the submission.

While revising your submission, please upload your figure files to the [Preflight Analysis and Conversion Engine](http://pace.apexcovantage.com/) (PACE) digital diagnostic tool.  PACE helps ensure that figures meet PLOS requirements. To use PACE, you must first register as a user. Then, login and navigate to the UPLOAD tab, where you will find detailed instructions on how to use the tool. If you encounter any issues or have any questions when using PACE, please email us at [figures@plos.org](mailto:figures@plos.org).

PLOS has incorporated [Similarity Check](http://www.crossref.org/crosscheck.html), powered by iThenticate, into its journal-wide submission system in order to screen submitted content for originality before publication. Each PLOS journal undertakes screening on a proportion of submitted articles. You will be contacted if needed following the screening process.

To resubmit, use the link below and 'Revise Submission' in the 'Submissions Needing Revision' folder.

We are sorry that we cannot be more positive about your manuscript at this stage. Please do not hesitate to contact us if you have any concerns or questions.

Yours sincerely,

Ian Henderson
Guest Editor
PLOS Genetics

Gregory P. Copenhaver
Editor-in-Chief
PLOS Genetics

Reviewer's Responses to Questions

**Comments to the Authors:
Please note here if the review is uploaded as an attachment.**

Reviewer #1: This manuscript characterizes the distribution of crossovers in an axr1 mutant as well as its methylome and reaches the conclusion they are uncoupled from one another. The crossovers in the mutant become much more distal compared to wild type and pairing is affected, yet telomeric bouquets are unaffected. Using whole genome bisulfite sequencing to explore the DNA methylation landscape in axr1 revealed hypermethylation of transposons especially in the non-CG context.

The data in the axr1 mutant support increased distal crossovers and hypermethylation of transposon sequences, however, given the generality of AXR1 function it seems these are two unrelated studies. The two distinct aspects of the study seemed forced together to make a conclusion that cannot be supported by the data. The results are interesting, but the conclusions should be modified as they as well as the title are not justified by the data. The comments listed below are intended to improve this study with a special emphasis on bisulfite sequencing analysis and interpretation.

Major comments:

1. The presentation of the WGBS data is unconventional and the methods section is lacking detail. How is methylation level determined in table S2 (i.e. C/C+T?)? Same for number of sites (this metric isn’t that useful). How is methylation level in figures determined?

Scatter plots (Fig 3A) and metaprofiles (Fig 3A, 4C, sup Fig S4B, S5) are very conventional ways to present WGBS data. The method section refers to our previous articles published in *The Plant Cell* last year where the methods are fully described. Trimming/mapping/DMR calls were all done with scripts extensively used by the community: Trim_Galore, Bismark, Bowtie and R/Bioconductor packages like *bsseq* or *DSS*.

All other details of methodology are described in the legends of the Figures and in particular Figure 3. The “methylation levels correspond to the ratios of methylated cytosines over the total number of cytosines.” was mentioned in the legend of Fig 3A, 4A and Supplementary Figure S5. The number of reads covering cytosines taken into account was mentioned in the Method section “Only cytosines covered by at least five reads were included to determine the average methylation levels.”.

The method used to call DMRs and the statistics were described in detail in *The Plant Cell* paper, however we have completed the current manuscript by including a brief description of the DMR calling procedure as follows:

“Differentially Methylated Regions (DMRs) were identified using the following R packages: bsseq version 1.7.7 [74] and DSS version 2.11.3 [75] as previously described [76] **using standard DSS parameters (DMR length > 50 bp, number of Differentially Methylated Loci > 3, more than 50% of C sites with p-value < 0.0001). DMRs closer than 50 bp were merged. To define hypo- or hyper-DMRs, we applied an additional cutoff to keep DMRs with at least a 10% change in methylation ratio for CHHs, 20% for CHGs and 30% for CGs.**”. In addition, we added the following information to the legend of Fig 3C: “DMRs correspond to regions showing, at least, a 10% change in methylation ratio for CHHs, 20% for CHGs and 30% for CGs”.

The metrics described in Table S2 were outputs given by the Bismark mapper. We additionally provided in parentheses the % of methylation per cytosine context, which we believe is very useful to evaluate the overall cytosine methylation states. We agree that the total number of cytosine methylated in the different contexts is less useful and we have removed these numbers.

The following legend was added to the Table: “The number of clean paired-end reads obtained after trimming, the number of mapped paired-end reads, the coverage, and the % of cytosines methylated in the different contexts are indicated. Bisulfite conversion rates were determined by aligning the reads to the chloroplast sequences of Arabidopsis (TAIR10).”

2. In general, there is a lack of context regarding the relationship between DNA methylation and crossovers. It’s my understanding that it’s not DNA methylation per se that affects crossovers but the chromatin it is associated with (i.e. heterochromatin/H3K9me2, etc). As a result, the title is inappropriate as there is no evidence presented that the chromatin landscape is altered, as the methylation variation seems to occur at regions that were previously methylated.
We agree and we have changed the title to the following one:

**“Meiotic crossover localization changes are uncoupled from the effects of MET1, CMT3, or POL IV when *AXR1* is inactivated in *Arabidopsis thaliana***”

3. For all of the metaplots, the authors should include heatmaps, to demonstrate whether there is new methylation occurring at new regions or if it is additional methylation occurring at the same sequences.
We demonstrated that regions hypermethylated in *axr1^-/-^* are TEs gaining methylation and not new regions. First, the TE metaprofiles of Fig 3B are in agreement with an increase of methylation in TEs that were already largely methylated in the wild type. Second, the hyperDMRs found between *axr1^-/-^* and the wild type were annotated and most of them overlap with methylated TEs as stated in the manuscript: “HyperDMRs mainly overlapped with TEs (44% of the CG hyperDMRs, 77% of the CHG hyperDMRs and 76% of the CHH hyperDMRs).”. Finally, CHG hyperDMRs are mainly found in the pericentromeric regions where most TEs are localised (Figure 3D).

For all these reasons, we believe that adding heatmaps to the metaplots is unnecessary and would reduce the figure readability.

4. Although averaging the data between the replicates reveals hypermethylation, especially in the non-CG context, the individual replicates presented alone do not always support this conclusion. Given the authors have replicate data, all replicates should be shown in all figures where appropriate.

Before pooling the results obtained with different biological replicates to present them in the figures, we first verified (1) that individual replicates were very similar between them (we now provide the clustering analysis for the different replicates using methylKit; new Supplementary Fig S4A) and (2) that the same conclusions were supported when replicates were pooled.

We have added the following sentence in the legends of the figures and supplemental figures wherever needed “The average methylation levels were determined by combining the biological replicates for each genotype”.

We now provide the metaprofile graphs showing results for all replicates for *axr1^-/-^* mutants and their corresponding WT controls, as well as for the *axr1-12* allele, in the supplement (new Supplementary Figure S4B). Graphs obtained with all individual replicates and pooled replicates support the same conclusions. Moreover, the new version of Figure 6A shows CG methylation levels for all *axr1^-/-^* and wild-type replicates.

5. Figure 3 demonstrates that hypermethylation is occurring in the heterochromatin enriched areas, which are regions where crossovers are suppressed. It shows that there are two unrelated processes ongoing here and makes it hard to understand the study as presented.
As stated in the original manuscript, methylation increases in all TEs whether they are located in heterochromatin or in gene-enriched area. Since heterochromatin is enriched in TEs, the most drastic effects of the *axr1^-/-^* mutation described in Figure 3 are observed in this region. However, the hypermethylation of TEs is general, and also occurs in regions where crossovers are not suppressed in the wild type (near genes). Our clustering analysis shows that the methylation increase following the gradient of TEs between heterochromatin and euchromatin.

In the manuscript, we claim that “aberrant CO localization pattern correlates with a drastic DNA hypermethylation of TEs in both meiotic and somatic cells, mainly in the non-CG contexts.”.

6. The *cmt3* mutant data shows exactly what is expected, if you lose the CHG methyltransferase you will lose CHG methylation. The *nrpd* data is odd as it is well known that hypomethylation occurs in this mutant especially in the euchromatic regions, however in the *nrpd1* mutant the CHH methylation is higher than many other genotypes including wild type. How is this possible?
The CHH methylation in both *nrpd^-/-^* and *axr1^-/-^* *nrpd^-/-^* double mutants are much lower than the wild type (Figure 6B), so there must be a misunderstanding here. This might be because the color of the *nrpd^-/-^* line was similar to the one of the wild type, and we have corrected this.

7. The last paragraph of the results needs to be rewritten as the comparison to met1 mutants and the mutants in this study are misleading. In met1 mutants, CG methylation is lost, but importantly there is a loss of chromatin structure as well (especially at heterochromatin). This does not happen in the nrpd1 or cmt3 mutants so the comparisons made should be clarified to make this clearer.
We agree, and we have clarified that aspect. Our point was to test separately the different DNA maintenance methylation contexts to confirm that methylation is broadly affected in *axr1^-/-^*. This is now explained more clearly in the last paragraph of the results as well as the hypotheses tested by combining *axr1^-/-^* with the individual *met1^-/-^*/*cmt3^-/-^*/*pol iv^-/-^* mutants.

Minor comments:

1. The black box in Figure 2A from an image program is odd. Remove it or use a different image.

We have redone Figure 2 with the two images in high resolution and we hope it will solve the problem.

2. The figure fonts/style etc are different between different figures. Many figures are in low resolution.

The resolution of Figure 3 has been improved. We have homogenized the styles between the figures.

3. WGBS conversion rates are not listed in Table S2.

Bisulfite conversion rates are now listed in Table S2.

4. The methods need details such that the study can be replicated.
We provide additional details in the Method section as well as in the legends of the different figures.

_________________________
Reviewer #2: In their manuscript entitled “Meiotic Crossover localization and DNA methylation are uncoupled in Arabidopsis thaliana when AXR1 is inactivated” Christophorou et al. extend upon their previous characterization of axr1 mutant phenotypes and investigate the connections between AXR1 and DNA methylation. First, the authors use genetics to combine axr1 mutants from different genetic backgrounds, enabling them to assess the effects of axr1 loss on crossover rates and distribution at a much higher resolution than in their previous work. While these experiments are quite elegant and do indeed provide a more detailed view of the effects of the axr1 mutant, they largely confirm previously reported trends--that in axr1 mutants the number of crossovers remain similar to wild-type but their distribution is altered such that they are more clustered and are relegated to the ends of the chromosomes. As these findings do not provide additional insights into the function of AXR1 in meiosis, they might be more relevant to a highly specialized audience. More interestingly, the authors find that in one axr1 mutant DNA methylation levels are altered. While this of potential interest, especially given previous connections between DNA methylation and meiotic recombination, it remains unclear if these changes in methylation are directly connected to the axr1 dysfunction as only one allele is assessed and no complementation data is provided. Furthermore, this change in methylation doesn’t appear to be connected to the crossover mislocalization effect observed in the axr1 mutants. Thus, to support their claims and elevate the impact of their work, additional experiments (see below) regarding the connections between DNA methylation and AXR1 are required.

Major Comments:

It is not clear what new information is provided in the “DSB initiation occurs normally in axr1-/- meiocytes” section. Perhaps the difference is the inclusion of a marker for H3K9 methylation? If so, this paragraph should be reworded to make this more clear. Alternatively, if no additional insights are provided, this data could be removed.
To explain the rational of the analysis we added the following sentence in the paragraph **“To confirm this result and to exclude that changes in CO location observed in *axr1^-/-^* were missed due to the 2D spread technique, we immuno-localised DMC1 on 3D-structure preserved meiocytes”**.

For the methylation analyses the following additional experiments and analyses should be included to determine what effect, if any axr1 mutants have on DNA methylation.
The bisulfite sequencing should be repeated using another Col allele (for example the one used in Jahns et al 2014) OR bisulfite sequencing using a line complemented with an exogenous AXR1 construct should be included. Without complementation or a second allele, one cannot exclude that the methylation defects are do to a second site mutation in a gene required for DNA methylation.

We clearly agree with this remark and we have therefore sequenced the methylome of another *axr1* allele (*axr1-12*; Tagliaro-Jahns, *PLoS Biology*, 2014) which is in the Col-0 background. The new sequencing results are presented in Sup Fig S4B (patterns of methylation for TEs and genes in all methylation contexts) and Figure 6A (boxplot showing the CG methylation levels in 100 bp-bins partitioning the whole genome). The methylome of *axr1-12* mutants is very similar to the methylome of all *axr1^-/-^* biological repeats.

Table S2 was completed with the sequencing statistics, and the raw BS-seq data for *axr1-12* are deposited under the same accession number as before (available from the ENA database; accession number PRJEB28434).

For the current (and additional) bisulfite sequencing data sets the replicates should be analyzed individually to determine the robustness the hypermethylation phenotypes as this would reveal if all the replicates show hypermethylation at the same sets of loci. The coverage of the current data should be sufficient to allow such overlaps to be conducted.

The average coverage is around 22. We have included the coverages for all individual replicates in the new version of Supplementary Table S2.

We now provide the graphs showing all replicates for *axr1^-/-^* mutants and their corresponding wild-type controls in the supplement (new Supplementary Figure S4). Graphs obtained with all individual replicates and pooled replicates support the same conclusions.

We also provide the clustering analysis for the different replicates, obtained with methylKit (new Supplementary Fig S4A), demonstrating that all replicates from the same genotype cluster together.

For Figure S8:
more clones (15-20 per genotype) should be sequence to draw conclusions from this data.
In addition, the single mutant controls should be included in this analysis otherwise its not clear which mutation is influencing the DNA methylation.
Finally, I am not sure what point is being made using this data as it is already well established that met1 mutants affect CG methylation. Please clarify.
We agree, and we have sequenced the complete methylome of the double *axr1*^-/-^ *met1*^-/-^ mutant and the *met1*^-/-^ F2 siblings to get a broader view of the methylation changes occurring in the *met1*^-/-^ backgrounds. The genome-wide results obtained are in agreement with the observations previously described at the *AtSN1* locus and shown previously in Supplementary Figure S8 (Methylation patterns of the *AtSN1* transposon) that was removed in this new version of the manuscript. The genome-wide patterns of methylation for *axr1*^-/-^ *met1*^-/-^ and the individual *met1*^-/-^ control are shown in the new Figure 6A and the results, showing similar patterns for CG methylation in both *axr1*^-/-^ *met1*^-/-^ and *met1*^-/-^, are now described in the new version of the manuscript.

Table S2 was completed with the sequencing statistics, and the raw BS-seq data for both *axr1*^-/-^ *met1*^-/-^ and *met1*^-/-^ mutants are deposited under the same accession number as before (available from the ENA database: accession number PRJEB28434).

Minor comments:

-) Typos in author summary
talso=(also
evnts=events
od=of
This was corrected.

-) Figure 1C, the black boxes on the x axis to denote the centromeres are missing
-) Figure 3G legend should refer to the clusters defined in F, not b.
-) Figure S4 the boxed regions showing the DMRs are missing.

Supplementary Figure S4C (which was previously Fig S4) has been improved. The boxed regions corresponding to DMRs are shown, the location of the regions within the whole genome is now provided.

-) For Figure S6 what are the scales for the Y axis?
These are densities of DMRs, so providing the scales would have no particular meaning.

_________________________
Reviewer #3: The overall premise of the Christophorou et al. manuscript is very attractive as factors controlling the distribution of crossovers (COs) are still poorly understood. The work is a follow-up of a study which found biased distribution of CO events along chromosomes in the axr1 mutant. However, the axr1 phenotype turns out to be quite complex. The mutant exhibits increased DNA methylation genome-wide but this does not appear to be directly associated with the CO distribution patterns. The over conclusion is quite interesting, implying that factors other than chromatin state strongly influence crossover distribution. However, the validity of this tenet may be questioned given how limited information the authors have on the actual DNA methylation patterns during meiosis.

The majority of the information on DNA methylation patterns that Christophorou et al. rely on comes from somatic cells. Taking into account the recent studies on the dynamic nature of DNA methylation during meiosis in Arabidopsis, how indicative are DNA methylation data from leaf tissue for what is happening in meiocytes? For the axr1 single mutant, the authors tried to confirm hypomethylation of meiotic chromosomes using immunolocalization, but this approach is very imprecise. Conducting bisulfite sequencing on meiocytes, or at least meiotic anthers, would clarify this issue.

We confirmed that DNA was hypermethylated in meiocytes using Antibodies directed against 5 methylcytosine. We agree that conducting bisulfite sequencing on meiocytes would allow a more precise analysis of the localization and the extent of DNA hypermethylation. This technique is only mastered by one laboratory and we cannot perform it for this study.

There is even more uncertainty with regard to the double mutant analyses. Does combining DNA methylation mutations with *axr1* indeed bring the DNA methylation patterns back to those of wild-type plants? Christophorou et al. examined DNA methylation by examining it at selected sites in the genome, predominantly TE regions. However, these are not the places where COs are generally formed. To be able to draw meaningful conclusions, the authors should conduct a full-genome analysis.
Calling DMRs between mutants and wild-type repeats is already a full-genome analysis because DMRs are obtained with no bias, in all contexts, taking into account the variation between all three biological replicates.

Importantly, combining the *axr1*^-/-^ mutation with *met1*^-/-^ or *cmt3*^-/-^ or *pol iv*^-/-^ mutations have a limited consequence on DNA methylation patterns observed in the individual *met1*^-/-^/*cmt3*^-/-^/*pol iv*^-/-^ mutants. In other words, double *axr1*^-/-^ *cmt3*^-/-^ or *axr1*^-/-^ *met1*^-/-^ or *axr1*^-/-^ *pol iv*^-/-^ mutants have nearly the same methylomes as individual *cmt3*^-/-^ or *met1*^-/-^ or *pol iv*^-/-^ mutants since there are very limited numbers of DMRs found between the double and the single mutants. This was mentioned in the original version of manuscript for both *cmt3*^-/-^ and *pol iv*^-/-^ backgrounds:

“Similar results were obtained when the methylomes of *axr1*^-/-^ *cmt3*^-/-^ and *cmt3*^-/-^ F2 plants were compared since a limited number of CHG DMRs was identified (0 hypoDMR and 40 hyperDMRs)”;

“The same results were obtained when we compared the methylomes of *axr1*^-/-^ *pol iv^-/-^* and *pol iv*^-/-^ mutant leaves. We found only 146 CHH hyperDMRs between *axr1*^-/-^ *pol iv*^-/-^ and *pol iv*^-/-^ and 5 CHH hypoDMRs.”;

and we have included the new data obtained by sequencing the *axr1*^-/-^ *met1*^-/-^ and *met1*^-/-^ methylomes:

“By comparing the methylomes of *axr1^-/-^* *met1*^-/-^ and *met1*^-/-^ F2 plants, a limited number of CG DMRs was identified (0 hypoDMR and 123 hyperDMRs), indicating that the CG methylation patterns of *axr1*^-/-^ *met1*^-/-^ and *met1*^-/-^ somatic cells are similar.”

To better described the differences between the double *axr1*^-/-^ *cmt3*^-/-^ or *axr1*^-/-^ *met1*^-/-^ or *axr1*^-/-^ *pol iv*^-/-^ mutants and *axr1*^-/-^, we determined the number of hyperDMRs found between the double *axr1*^-/-^ *cmt3*^-/-^/*met1*^-/-^/*pol iv*^-/-^ mutants and their individual *cmt3*^-/-^/*met1*^-/-^/*pol iv*^-/-^ backgrounds, coming from the original *axr1*^-/-^ background. 12% of the CG DMRs found between *axr1*^-/-^ *pol iv*^-/-^ and *pol iv*^-/-^ are also found in the *axr1*^-/-^ background compared to Col-0, 23% for *axr1*^-/-^ *cmt3*^-/-^ and 31% for *axr1*^-/-^ *met1*^-/-^. 61% of the CHG DMRs found between *axr1*^-/-^ *pol iv*^-/-^ and *pol iv*^-/-^ are also found in the *axr1*^-/-^ background compared to Col-0, 28% for *axr1*^-/-^ *cmt3*^-/-^ and 53% for *axr1*^-/-^ *met1*^-/-^. 2% of the CHH DMRs found between *axr1*^-/-^ *pol iv*^-/-^ and *pol iv*^-/-^ are also found in the *axr1*^-/-^ background compared to Col-0, 14% for *axr1*^-/-^ *cmt3*^-/-^ and 6% for *axr1*^-/-^ *met1*^-/-^. Therefore, combining DNA methylation mutations with *axr1*^-/-^ bring the DNA methylation patterns back to those of the individual *met1*^-/-^/*cmt3*^-/-^/*pol iv*^-/-^ mutants with some few exceptions. This is now stated in the manuscript.

Finally, the overall conclusion presented in the title that CO localisation and DNA methylation are uncoupled in the axr1 mutant is also uncertain. Have the DNA methylation mutations used in the study completely obliterated DNA methylation in the mutants? DNA sites that are critical for producing specific recombination patterns may still be methylated in the mutant even if there is indeed a global reduction of DNA methylation. A more precise conclusion would be that CO localization in axr1 becomes uncoupled from the effects of the MET1, CMT2, and POLIV genes.
As mentioned above, we agree that some loci remain methylated in the double mutants compared to the individual *cmt3*^-/-^/*met1*^-/-^/*pol iv*^-/-^ mutant backgrounds but their number is limited compared to the original number of DMRs found in *axr1*^-/-^ and the DNA methylation patterns of *axr1*^-/-^ are similar to the double mutant obtained by combining *axr1*^-/-^ with DNA methylation mutant. However, we agree that not all *axr1*^-/-^ hyperDMRs are abolished in the double mutant and we have therefore added the following sentence to the Result section to smooth our claim:

“Altogether, the results indicate that mutations affecting methylation are epistatic over *axr1*^-/-^ in somatic cells, **although we note that a few amount of DMRs found in the *axr1*^-/-^ background are still detected in the double *axr1*^-/-^ *met1*^-/-^ (0.6 % of the *axr1*^-/-^ CG hyperDMR), *axr1*^-/-^ *cmt3*^-/-^ (7 % of the *axr1*^-/-^ CHG hyperDMR) and *axr1*^-/-^ *pol iv*^-/-^ (1.6 % of the *axr1*^-/-^ CHH hyperDMR**).”

Other major issues:
Page 6: Can the authors use some kind of a statistical measure to assess the degree of clustering? Otherwise, it is a bit difficult to define how strong this phenotype actually is.
The clustering has been analyzed by measuring the physical distances between 2 COs on the same chromatid. We show in Figure 1B that the distribution of distances between two COs is significantly different in *axr1^-/-^* compared to wild type. Double COs are predominantly close to each other in the mutant whereas in the wild type they are separated by half a chromosome in average. We also showed that both distributions are significantly different from random. We think that it is the most accurate way to show that COs tend to cluster in the mutant. In addition, Figure 1D shows that COs tend to be closed to the telomeres in the mutant whereas they are distributed all along the chromosomes in the wild type. We combined both analyzes to suggest that COs cluster tends to occur in the distal regions of the chromosomes

Page 8: Is chromosome pairing abolished in the axr1 mutant or just delayed? If the latter, perhaps the defect is in the speed of meiosis progression rather than chromosome interactions.
We know that chromosome interaction is highly disturbed in *axr1*^-/-^. The absence of full synapsis was reported in Jahns *et al*., 2014 and here, we report pairing defects at both centromeric and telomeric regions during meiosis (Figure 2). It does not exclude that there is also a defect in the speed of meiosis progression even if it never produces fully synapsed chromosomes

Page 14/line 5: since the authors only assess distribution of DSBs by a visual assessment of DMC1 foci on immunolocalization images, the statement that the axr1 mutation does not affect DSB formation should be tone-down. Alternatively, can the authors measure spacing of DMC1 foci?
In Arabidopsis, we do not have an accurate way to measure directly the rate of DSB formation. Thus, DMC1 and/or RAD51 foci in meiosis serves as a proxy to localize DSB repair sites and thus indirectly to look at DSB sites. Our 3D experiment tells us that there are no large regions devoid of DMC1 foci. The spacing of DMC1 foci is something that we are working on but it is not an easy task and we haven’t got yet the tools to do it. Thus we modified the title of the paragraph : “**No difference in early recombination events can be detected between wild type and *axr1***^-/-^”.

Page 16/line 2: Similarly, I’m not sure that visual assessment of DAPI staining patterns is indeed a good marker of chromosome structure. Can the authors confirm this conclusion by conducting specific measurement of the DAPI-stained regions?
In S7 Fig, we quantified the length of the DAPI dense regions in wild type (n=118) and in *axr1*^-/-^ meiocytes (n=138). To be more accurate this length was divided by the nucleus area. The same approach was used for the Nucleolar Organizer region (NOR). In both cases we did not detect a significant difference.

Page 16/line 15: But the study of Underwood et al. seems to contradict this statement…
In Underwood *et al*, in the *cmt3*^-/-^ background, there is a clear increase of CO formation in the centromeric region of chromosome 3 observed by FTL. When CO distribution is analyzed genome-wide, this increase is confirmed but other centromeric regions like those of chromosome 1 or chromosome 2 do not contain significantly more COs. Thus, the correlation between CO localization and DNA methylation is far from being straightforward. This the reason why we suggest in the next sentence that in the *axr1^-/-^* background, when DNA methytransferase pathways are compromised, even if DNA is hypomethylated, chromatin could still be modified impairing the proper localization of COs.

Page 15/line 21: Is it really true that “DNA methylation down-regulates CO formation…” given that some studies reported decreases of CO numbers in pericentromeric regions of chromosomes in Arabidopsis CG methylation mutants.
As mentioned above and in the Discussion, the relationships between DNA methylation and CO localization are far from being straightforward. Depending on the DNA methylation pathway that is inactivated and the region analyzed, COs are either slightly increased or slightly decreased or not significantly changed. As mentioned above when CMT3 is inactivated there is a slight increase in CO formation in some centromeric regions but not all of them. In the work that the reviewer mentioned, in the *met1*^-/-^ background, COs are slightly increased in the chromosome arms and in the centromeric regions coupled to a slight decrease in pericentromeric regions. This has been interpreted as a remodeling effect due to interference. To underline the complexity of the relationships between DNA methylation and CO localization we mentioned that **“DNA methylation down-regulates CO formation…in some genetic backgrounds**”.

Minor issues:
Line 14 of abstract: use “limiting factor” instead of “limitator.”
Corrected.

Lines 10 and 14 of Author Summary: use “localization change” instead of “delocalization.”
Corrected as well as in other parts of the manuscript.

Line 12 of Author Summary: use “changes of the pattern” instead of “modification in the pattern.”
Corrected.

Page 1/line 3: use “DNA replication” instead of “replication.”
Corrected.

Page 1/line 25: it would be good to distinguish between species with small genomes, such as Arabidopsis, in which pericentromeric regions are small, and large genome-species, such as barley or wheat, in which the pericentromeric regions are very extensive.
We agree with the reviewer and that is the reason why in page 2 we have a paragraph dedicated to barley as an example of plants with large genomes.

Page 2/line 4: [8] is a wrong citation
Corrected.

Page 2/line 24: “DSBs appear gradually along the genome” – in which species?
Corrected. “In *S. cerevisiae* or in barley …”.

Page 3/lines 1-3: “In both human and barley….” But citation [18] only refers to barley.
Corrected. A citation referring to Human meiosis has been added.

Page 3/line 15: since the “chromosome mobility machinery” has not been studies in Arabidopsis, this statement may be premature.
Corrected. We changed the sentence. In Arabidopsis, SUNs proteins and PSS1, a Kinesin1-like protein, that are homologs of proteins known to be part of the chromosome mobility machinery in yeast, worms and mammals*,* are both required for meiotic CO control.

Page 4/lines 1 – 2: use “cytosines methylated at non-CG sites” instead of “non-CG methylated cytosines.”
Corrected.

Page 6/line 24: telomere bouquet instead of “bouquet telomeres.”
Corrected.
