# Supplementary material for: AXR1 affects DNA methylation independently of its role in regulating meiotic crossover localization
Source: PLoS Genet. 2020 Jun 29;16(6):e1008894. doi: 10.1371/journal.pgen.1008894 (PMC7351236; doi:10.1371/journal.pgen.1008894)
Supplement: Attachment — Submitted filename: ResponseCHristophorou_24042020.docx [file pgen.1008894.s017.docx]

Response to the referees:

Reviewer #1: I appreciate the authors attempt to improve aspects of this manuscript. However, there are still a number of suggestions from my original review that have not been sufficiently addressed.

Table S2 is still quite unconventional to the field although it has improved. Most labs are not interested in the percent of cytosines methylated, as these authors have reported in table S2. Although this can be a useful metric, the authors should include the methylation level for each context in addition to percent of methylated cytosines.

We have removed the percent of cytosines methylation from Table S2. Those were the numbers given by the bismark software without any normalization between samples. The manuscript contains all the data describing the differences of methylation patterns in the *axr1* mutant for the three contexts, including proper analyses with filtering and normalization (Fig 4, 5, 6 and Sup Fig S5-S10). We beleive that providing the raw percent of methylation does not add much to the Table S2.

I find the new title odd. In addition to CO localization being uncoupled from MET1, CMT3, etc it's uncoupled from LOTS of genes. As i stated in my original review, this is a classic pleiotropic mutant. The methylation phenotype occurs independent of the cross over phenotype, yet they are forced together in this study. Rublyation/neddylation likely affects a lot of other proteins, which is why these two distinct phenotypes exist. A more accurate title should be included in a new version of this manuscript.

We have changed the title to the following one: **Inactivation of *AXR1* impairs meiotic crossover localisation and transposon methylation in *Arabidopsis* *thaliana***

The heatmaps are an integral component of the results. Metaplots will not address if regions that are already methylated become more methylated as it is a single value that collapses all features into a single line. When the line on a metaplot shifts up or down it clearly indicates a change. However, it is unclear if the change is due to new regions becoming methylated/demethylated or existing regions that are methylated gaining/losing methylation or a combination of both. Incorporating heatmaps provides this exact information. The can be included as supplemental data if the authors feel it would interfere with the main figures.

Metaplots for DMRs also indicate whether the regions were methylated in the WT, we have therefore included these data. In addition, we have included the heatmaps as requested by the referee. Both DMR metaplots and heatmaps are now included in the supplement (Sup Fig S7 and S10). They all show that hypermethylated regions of *axr1* were already methylated in the wild type, confirming that TEs gain methylation in the mutant.

This statement is not useful, "aberrant CO localization pattern correlates with a drastic DNA hypermethylation of TEs in both meiotic and somatic cells, mainly in the non-CG contexts.” DNA hypermethylation occurs genome-wide. As the authors replied in the previous statement, hypermethylation occurs everywhere. If you take their results as presented, that hypermethylated regions are occurring at previously methylated regions, then the correlation between CO localization and non-CG contexts is just a correlation of TE density. The methylation has nothing to do with COs, at least based on the data presented in this study.

We have never claimed otherwise. We uncouple TE hypermethylation from CO formation when *axr1* is crossed with mutants specifically impaired in DNA methylation pathways, meaning that TE hypermethylation is not directly responsible for CO aberrant localization in *axr1^-/-^*. This is an important result as it disconnects DNA methylation from CO change of localization in the *axr1^-/-^* mutant background. We have therefore tried to smooth all sentences in the manuscript that would be ambiguous.

I don't doubt the results presented in this study, but there is a need for significant improvement to the clarity of the study. More careful interpretation of the results would also benefit the readers and the relevant research fields.

We never pretended that TE hypermethylation drives CO localization in *axr1^-/-^*. Indeed, we show the opposite, implying that methylation is probably not responsible for CO localization changes and disconnecting methylation from meiosis in this mutant. We believe that this is an important result for researchers working on meiosis or DNA methylation. Having said that, we understand the point raised by the referee and some parts of the paper were modified for clarity.

Reviewer #2: In the revised manuscript, Christophorou et al dealt with many of my more minor concerns, but unfortunately did not include any controls (i.e. WT) for their DNA methylation analyses using a second axr1 allele. Since the observed hypermethylation phenotype is quite subtle, and there can be variations in methylation levels between biological replicates, the lack of a WT control makes the new data hard to interpret. Thus, my concerns regarding the connections between AXR1 and DNA methylation remain. Without connections to DNA methylation the remaining data is largely descriptive and confirmatory of previous studies conducted at lower resolution.

The wild-type control sequenced with *axr1-12* is now included (orange lines in Sup Figure S5) and confirms the differences observed between all three *axr1-1* repeats and their corresponding wild type. This was not included in the previous version of the manuscript because the wild type sequenced together with *axr1-12* presents the same patterns of methylation as all other wild type shown in this study compared to *axr1* mutants sequenced at the same time. To gain further into the analysis of the AXR1 dependent DNA methylation, we have added the data of the DNA methylome performed in *axr1^-/-^* in meiocytes. This analysis confirms a deregulation of the dynamic of DNA methylation in *axr1*^-/-^. We agree with the reviewer that our work is descriptive but we disagree with the fact that our results confirm previous studies. Indeed, we disconnect DNA methylation from CO localization in *axr1^-/-^* whereas in other genetic backgrounds (*met1*, *cmt3*), COs localization can be slightly modified (reference within the manuscript).

Reviewer #3: The revised manuscript is much improved over the original version. However, I find two major issues remaining:
(i) Are DNA methylation patterns in leaves an appropriate proxy for the methylation patterns in reproductive tissues? I think this issue is critical, given that DNA methylation patterns are known to be dynamic during plant development and the fact the that the overall conclusion of study that CO patterns are uncoupled from the methylome patterns defies expectations. I feel the authors should do bisulfite sequencing from anther DNA or at least from flower buds at appropriate stages.

We sequenced the methylomes of both wild-type and *axr1^-/-^* male meiocytes. We found that DNA methylation at chromosomal level was similar in both the CG and CHG contexts between the wild-type and *axr1^-/-^* male meiocytes, but was increased for CHH in the centromeric and pericentromeric regions of *axr1^-/-^* (Fig 4A, S6A Fig). The average gene methylation was similar in *axr1*^-/-^ and wild-type meiocytes (Fig 4B, S6B Fig). For TEs, no differences were observed for both CG and CHG methylation levels, however, the CHH methylation of *axr1*^-/-^ TEs was increased in *axr1*^-/-^. These data have been added to the manuscript.

(ii) I still have an issue with the way the authors have compared DNA methylation levels among genotypes. I understand that the axr1 mutants show hypermethylation at many sites across the genome. But are these sites relevant for CO formation? I think the authors should specifically examine the sites identified as harboring COs in wild-type plants.

We cannot compare directly the crossover map and the DNA methylation data because the scale of analysis is different. The genetic data give a resolution at an average of 1.2 Megabases whereas potentially each cytosine has been looked at with the methylome sequencing. In the *axr1^-/-^* background, our data suggest that DNA methylation does not play a direct role on the crossover distribution. This result does not imply that it is the case in others genetic backgrounds. It is likely that the inactivation of the neddylation pathway has pleiotropic effects and that other factors, acting on the chromatin structure, may have a more direct role on CO localization. We have tried to clarify this issue in the manuscript.
