# Supplementary material for: AXR1 affects DNA methylation independently of its role in regulating meiotic crossover localization
Source: PLoS Genet. 2020 Jun 29;16(6):e1008894. doi: 10.1371/journal.pgen.1008894 (PMC7351236; doi:10.1371/journal.pgen.1008894)
Supplement: Attachment — Submitted filename: Christophorou re-review.docx [file pgen.1008894.s018.docx]

This revision provided additional data, in the form of key controls, that strengthen the authors claims regarding the effects of axr1 mutants on DNA methylation patterns. Regarding this new data I have several additional comments as detailed below. Overall, as was mentioned by another reviewer, the data are now more clear, but the messaging at times could be more transparent about the connections (or lack thereof) between the different phenotypes observed in the axr1 mutant. I have provided feedback related to this point as well.

DNA methylation analysis:

1. In Table S2, please make it clear which samples and controls were grown and prepped together. In the response to reviewers, it is stated that axr1-12 and presumably WT rep#4 were done in parallel, but this is not clear in the table.
2. In Figure 6A, please add the WT control for axr1-12. Also, please clarify why this data is shown as a boxplot rather than a metaplot to match the other panels in the figure.
3. In Figure S5A please add the WT control and axr1-12 to the clustering analysis.
4. In Figure 4A, metaplots are used to show the differences between the mutant and WT methylation levels across the chromosomes, where it is clear the hyper CHH is in the centromeric regions, but in Fig 5a differences are shown with scatter plots. For consistency it might be useful to represent this data from two different tissues in the same manner, or show both types of plots even if some of the data is in the supplement.
5. It is standard practice to compare the methylation patterns of two alleles at a finer scale than presented here. Based on the metaplot analyses, both axr1 and axr1-12 show hypermethylation around the centromeres, but do they show hypermethylation at a similar set of genomic loci? Please identify DMRs in the axr1-12 mutant and provide a comparison with those identified for axr1. Assuming there is a high degree of overlap the methylation patterns, the WT and axr1-12 tracks could be added to the screen shots shown in Fig S5C.
6. Please reference and indicate in the methods what data sets were used to compare the axr1 DMRs to those in met1, cmt3, kyp, and poliv mutants.

Text comments:

1. Perhaps a title that more clearly conveys the conclusions of the work would be “AXR1 affects DNA methylation independent of its role in regulating meiotic crossover localization”?
2. The running title could also be modified to better reflect the data
3. Page 2. Its not clear what by the statement that axr1 affects “general methylation” in a plant? What is general methylation? Please be more specific.
4. Several times the authors state the methylation changes as “massive” or “drastic”, but no value is provided. Such a statement should either be pared with an empirical value (i.e the average % increase in methylation over the WT) and a reference to other work showing this change is very large compared to other mutants, or such adjectives should be removed.
5. In the intro the authors conclude that AXR1 acts to limit DNA methylation pathways. The data do support this conclusion, but do not provide information on whether this is a direct or indirect effect of AXR1 function. The text should be modified to make this clear.
6. In the author summary, the last sentence mentions “methylation of nucleic acids”. Since other bases can be modified, but this work is specific to cytosine methylation, the text should be modified to make this clear.

Minor comments:

- Figure 1B is referenced out of order, please reorder the text or figure.
- In the main text, page 8 line 25, the authors refer to 15 intervals with different CO rates. On Fig 1C, I count 18 asterisks (*) and there are also appear to be 18 regions in table S1. Please clarify.
- As the text referring to Fig 1B uses Mb units rather that % of chromosome length, perhaps the graph could be replotted or the text edited to allow this figure to be more easily interpreted.
- The Y axis label for Fig 2B is cut-off.
- In Figure S6 I believe the data is from meiocytes based on the legend, but they are labeled as “sc” which I presume stands for somatic cells. Please clarify.
- It is confusing that the supplemental data for the meiocyte and somatic tissues are mixed between Fig S5 and S6. I might be helpful to group the meiocyte and somatic data into separate figures. Indeed, as there are just two replicates and the metaplots are not too complicated perhaps fig S6 could be removed and in Fig 4 both replicates cold be shown?
- In Fig S7 several of the methylation scale bars for the heatmaps are overlapping the Y axis labels for the metaplots. Also, please indicate in the legend if the order of TEs is the same for the all the CG, CHG, and CHH heatmaps or whether they were ordered separately based on a specific parameter in each heatmap.
- I could not find Fig 7 or Fig S1-3 in the revised version. I assume they are not changed from the first revision. If otherwise, please clarify.
